# Elucidation of the ATP7B N-Domain Mg^2+^-ATP Coordination Site and Its Allosteric Regulation

**DOI:** 10.1371/journal.pone.0026245

**Published:** 2011-10-27

**Authors:** Claude Hercend, Cyril Bauvais, Guillaume Bollot, Nicolas Delacotte, Philippe Chappuis, France Woimant, Jean-Marie Launay, Philippe Manivet

**Affiliations:** 1 APHP, Hôpital Lariboisière, Service de Biochimie et de Biologie Moléculaire, Paris, France; 2 INSERM U942, Biomarqueurs et Insuffisance cardiaque, Hôpital Lariboisière, Paris, France; 3 Division of Structural Biology, Bioquanta, Paris, France; 4 APHP, Hôpital Lariboisière, Service de Neurologie, Paris, France; 5 INSERM U829, SABNP Laboratory, Evry, France; 6 Université Evry Val-d'Essonne, Evry, France; University of Cambridge, United Kingdom

## Abstract

The diagnostic of orphan genetic disease is often a puzzling task as less attention is paid to the elucidation of the pathophysiology of these rare disorders at the molecular level. We present here a multidisciplinary approach using molecular modeling tools and surface plasmonic resonance to study the function of the ATP7B protein, which is impaired in the Wilson disease. Experimentally validated *in silico* models allow the elucidation in the Nucleotide binding domain (N-domain) of the Mg^2+^-ATP coordination site and answer to the controversial role of the Mg^2+^ ion in the nucleotide binding process. The analysis of protein motions revealed a substantial effect on a long flexible loop branched to the N-domain protein core. We demonstrated the capacity of the loop to disrupt the interaction between Mg^2+^-ATP complex and the N-domain and propose a role for this loop in the allosteric regulation of the nucleotide binding process.

## Introduction

**Figure 1:**
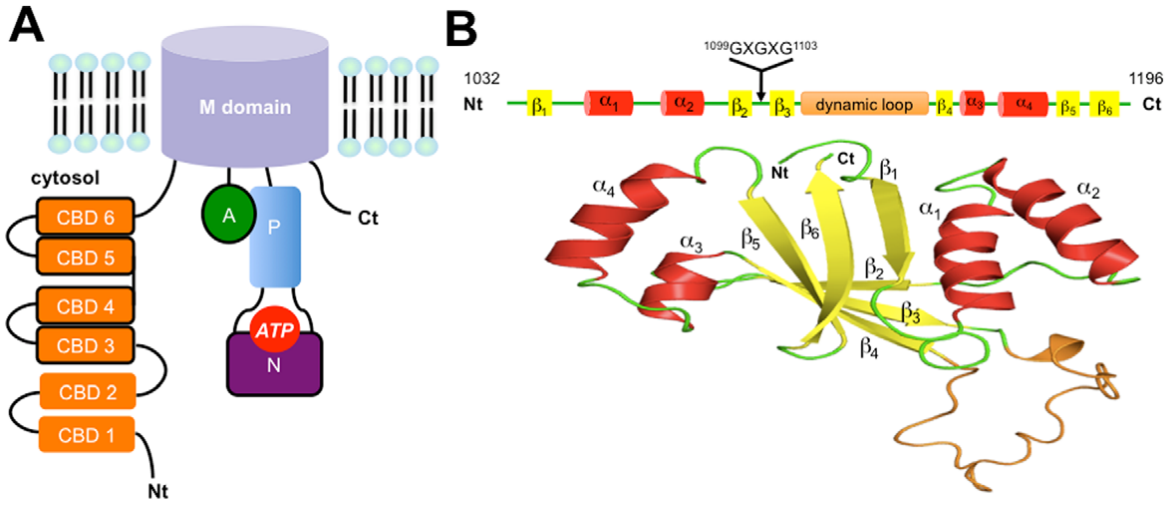
General organization of the ATP7B protein. (A) The different domains of the protein are presented: the six Copper Binding Domains (CBD), the transmembrane domain (domain M), the Actuator domain (A), the Phosphorylation domain (P) and the Nucleotide-binding domain (N). The different domains, which have been structurally characterized, are framed in black. (B) Sequence and structure available for the N-domain. Secondary structures and the dynamic loop are shown along with the position of the poly-glycine loop (GXGXG).

The Wilson disease (WD, OMIM 277900) is due to mutations in a copper transporter gene coding for the protein ATP7B that belongs to the P-type ATPase superfamily [1]. It is a rare and serious inherited sickness with the main clinical manifestations resulting in a systemic copper accumulation (liver, brain…). The incidence of this autosomic recessive disease is estimated at one for 30,000 to one for 100,000 individuals depending on the ethnicity of the affected population [2]. With a wide clinical spectrum and a slow progressive evolution, since liver will tolerate copper accumulation, early clinical diagnosis of WD remains difficult. This explains why patient condition is so serious when symptoms appear, like liver disease such as cirrhosis, neurologic disturbances or even psychiatric signs [3]. The standard diagnostic is based on the exploration of copper metabolism (copper, caeruloplasmin…) and molecular analysis of ATP7B gene mutations. The genetic diagnosis is difficult and may sometimes be a bottomless problem since WD has a marked genetic heterogeneity and most of the affected individuals are compound heterozygotes. Indeed, with a gene coding region of 4.3 kb and 21 exons, a full PCR amplification is out of reach in daily laboratory routine. Besides, more than 300 different mutations and 100 genetic polymorphisms have been published so far [4]. Even though some mutations are more frequent depending on the tested population, e.g. H1069Q in Caucasians [5] and R778L in Asians [6]. The pathogenesis of the disease is better understood since the discovery of the culprit gene (*Atp7b*) in 1993 [7]. The function of the protein in copper metabolism has been studied by using biochemical assays [8], cellular (hepatocytes) and *in vivo* models to decipher its cellular trafficking that mediates the export of the ion in different organs [9]. Human ATP7B is a copper ATPase (P35670) that shares the general domain organization of P-ATPases (Figure 1A). This transmembrane protein contains four domains with both N- and C-terminal ends located in the cytosol. An original metal binding site is composed of six distinct Copper Binding Domains (CBD) located in the N-terminal cytosolic part of the protein. The other domains are the Actuator domain, the Phosphorylation domain (P-domain) and the Nucleotide-binding domain (N-domain). This latter domain holds the ATP binding site and plays a major role in the catalytic cycle (Figure 1B). Experiments have been dedicated to the study of the impact of ATP7B mutations. Alterations of the molecular integrity of the ATP7B are responsible of a partial or total loss of function. For instance, yeast complementation assays have been successfully used to study ATP7B function. Despite being time consuming and highly skilled techniques, they allowed a deeper understanding of the pathogenic impact of mutations identified in different regions such as ATP-binding region composed of the N- and P-domain [10].

In parallel, structural studies concerning different domains of the ATP7B protein have been published. The N-domain structure studied in the present work was first obtained by NMR [11], followed by CBD5 and CBD6 [12]. Further studies led to the elucidation of CBD3 and CBD4 structures [13]. More recently, the Actuator domain (A-domain) of ATP7B was solved by heteronuclear NMR spectroscopy [14]. ATP is crucial for ATP7B function since phosphorylation initiates conformational changes in ATP7B that promote copper transport. The topology of the ATP binding site remains to be precisely defined despite NMR data available of the N-domain without and with ATP showing the existence of chemical shifts signals between ATP and protein residues belonging for instance to a poly-glycine loop [11]. However, Mg^2+^ ion is always absent from all structural and molecular modeling studies [11], [15], [16] despite the experimental demonstration of its great participation to the N-domain ATP affinity [17]. Most of all, attempts were made to clarify ATP/N-domain interactions without considering the role of the ion. Therefore, no clear nucleotide-binding site could have been proposed. The N-domain of the human ATP7B protein bears an intriguing long loop (A1114 - T1143) connected to the poly-glycine loop, which is absent in the ATP binding domain of other proteins of the P-ATPase family (SERCA, ZntA, CopA…) [18]. All the above observations, together with currently available unconvincing genotype-phenotype correlation data [19], call for the emergence of alternative methods such as molecular modeling, to investigate WD mutants with a clear protocol and highly accurate tools.

In the present study, the wild type (WT) N-domain structure was investigated by molecular dynamics (MD) simulations. The 3D structure of the apoform of the ATP7B N-domain [11] was used as a starting point to characterize the amino acids involved in the nucleotide binding of the wild type protein (WT). By means of 3D structural alignments and MD simulations validated by binding affinity measurements, an original ATP binding site is proposed where Mg^2+^ ion is hexacoordinated by the nucleotide and carboxylate residues of protein. The *in silico* 3D model proposed here also provides answers to the controversial issue on the magnesium role in the process of ATP binding as well as its impact on the dynamic of the N-domain. We propose a mechanism for the transition of the nucleotide between two binding modes (+/− Mg^2+^) with the allosteric participation of a long protruding loop.

## Results

### Elucidation of the N-domain Mg^2+^-ATP coordination site

Before interpreting the structure/function relationships of Wilson disease mutations and studying Mg^2+^-ATP binding, the first step has been the generation and experimental validation of the 3D models for ATP7B. The NMR structure of the N-domain has been solved without ATP coordinates, the precise binding site of ATP being still under debate. Experimental NMR data is available that could help to locate the binding site. Signals have been identified between ATP and G1099, G1101, G1149 and N1150 residues, suggesting a close proximity with ATP [11].

Compared to other P-ATPases, structural information is lacking for the P_Ib_-ATPase subtype concerning the direct interaction between the N-domain and ATP molecule [20]. This emphasizes the question about the definition of the ATP coordination site of ATP7B N-domain as well as the role of magnesium and the two glycine amino acids (G1099, G1101) previously mentioned. These two glycines, which are conserved in the P_Ib_ subtypes, belong to a region mentioned as a poly-glycine loop (^1099^GXGXG^1103^). This region has already been considered to be important in nucleotide binding to the Zn^2+^ transporting ATPase (ZntA) [21].

**Figure 2:**
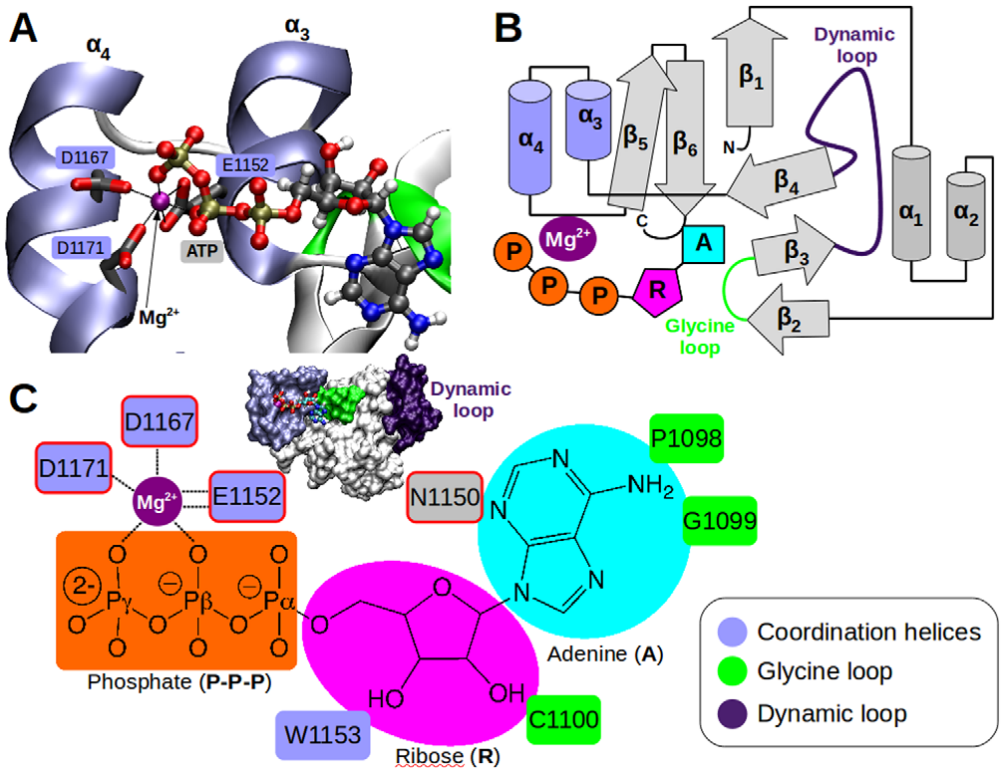
Mg^2+^-ATP coordination site within the N-domain (WT-Mg-ATP complex). (A) The surface of the protein is displayed with the main regions implied in interactions with ATP that are colored following the internal text label. A close view of the binding pocket is presented with α_3_/α_4_ helices (light blue) in ribbon representation with amino acid side-chains and ATP, with Mg^2+^ ion (purple sphere), in ball and stick representation. Carbon, oxygen, nitrogen, and phosphorous atoms are colored in gray, red, nitrogen, and orange, respectively. (B) Topological diagram of the secondary structures of the N-domain. Cylinders and arrows represent α-helices and β-strands, respectively. The nucleotide is shown with three different parts: Adenine (blue), Ribose (magenta) and Phosphate tail groups (orange). For illustration, glycine and dynamic loops are colored in green and purple, respectively. (C) 2D representation of the singular amino acids involved in direct interaction with magnesium ion or ATP atoms. Amino acids framed in red have been mutated (see Experimental section).

We first identified the N-domain residues capable of interacting with Mg^2+^. According to the statistical analysis performed with a bioinformatics database, the most probable residues able to coordinate Mg^2+^ are aspartic (33%) and glutamic (18%) acids (Figure S1). Then, we refined this analysis by mapping the N-domain protein surface with a spherical pharmacophore especially designed for respecting the specific geometrical and chemical constraints for an ideal Mg^2+^-ATP binding site (see Methods section). The Figure S2 shows the result of the pharmacophoric search that reveals a unique possible Mg^2+^ coordination site. This site is located in a region of the protein carrying a “DDE” structural signature between α_3_ and α_4_ helices involving side-chain oxygen atoms of residues E1152, D1167 and D1171 (Figure 2A). In addition, the Mg^2+^ binding site proposed is located nearby the region known for participating to ATP binding (G1099, G1101, G1149 and N1150).

To validate first the magnesium coordination proposed here and prior to the interpretation of the nucleotide binding effect studied by MD simulations, structural analysis of binding structural analysis of binding sites has been performed. More than 300 nucleotides binding sites using Mg^2+^ ion and ATP have been analyzed (see Methods section). The presence of aspartic and glutamic residues in these Mg^2+^-ATP binding sites reinforces the validity of the “DDE” motif used for positioning the magnesium ion (Figure S3).

### Experimental validation of the Mg^2+^-ATP coordination site

The contribution of the different regions of the protein taking part in the interactions with the nucleotide (phosphate tail, ribose and adenine moieties) was “tested” experimentally. The aim was to validate the binding site proposed through binding measurements of the nucleotide with Wild Type (WT) and mutated forms of the N-domain (Table 1). It shows first that the presence of the magnesium ion increases the nucleotide affinity of more than 30% for the N-domain. The dissociation constant (K_d_) for the WT decreases from 74.25±2.52 µM (ATP alone) to 51.94±2.79 µM (ATP, Mg^2+^). The substitution of any of the three residues of the “DDE” motif coordinating the Mg^2+^-ATP complex with an alanine (E1152A, D1167A or D1171A) significantly decreases the affinity for the nucleotide. The increase of the K_d_ ranges from 33% (D1171A) to more than 146% (E1152A) for Mg^2+^-ATP. Mutation of E1152A has the most marked impact on the ATP and Mg^2+^-ATP bindings. This may suggest a major role for the E1152 residue in the stabilization of the nucleotide. However, single point mutations do not prevent, if they markedly reduce, neither ATP nor Mg^2+^-ATP binding. The role of these “DDE” residues is evidenced by the absence of specific Mg^2+^-ATP binding to the N-domain (K_d_>1 mM) for the triple mutant (E1152A+D1167A+D1171A). It is interesting to note that this triple mutation also prevents ATP binding. To prepare the positioning of the nucleotide in the molecular modeling study, the N1150 was mutated to alanine (N1150A). This residue has been described previously as “close contact” with ATP. As for the others mutants, N1150A shows an increase in ATP binding affinity in presence of Mg^2+^ (17%). Compared to the WT, there is a slight perturbation of the ATP binding evidenced by the 11–33% drop of the K_d_ (+/− Mg^2+^).

**Table 1:** Nucleotide binding affinity for the N-domain of Wild Type and mutant proteins.

N-domain	*K_d_ (µM)*	
	ATP	ATP, Mg^2+^
**Wild-type (WT)**	74.25±2.52	51.94±2.79
**E1152A**	174.31±3.82**	128.84±2.74**
**D1167A**	96.92±3.91**	80.96±2.52**
**D1171A**	81.12±3.16*	68.04±2.71*
**E1152A+D1167A+D1171A**	no binding**	no binding**
**N1150A**	82.74±4.16*	68.33±2.82*
**Δ1121–1137**	no binding**	no binding**
**H1069Q**	76.52±1.72	54.37±1.66

The affinity is presented here via the dissociation constant K_d_ for the complex: N-domain- Mg^2+^-ATP (left column) and N-domain/ATP (right column). The amino acids belonging to the coordination helices are highlighted in light blue, the deletion of the dynamic loop is shown in purple.

No binding (K_d_>1 mM),

*P<0.05,

**P<0.01.

### MD simulation study of the WT-Mg-ATP complex

**Figure 3:**
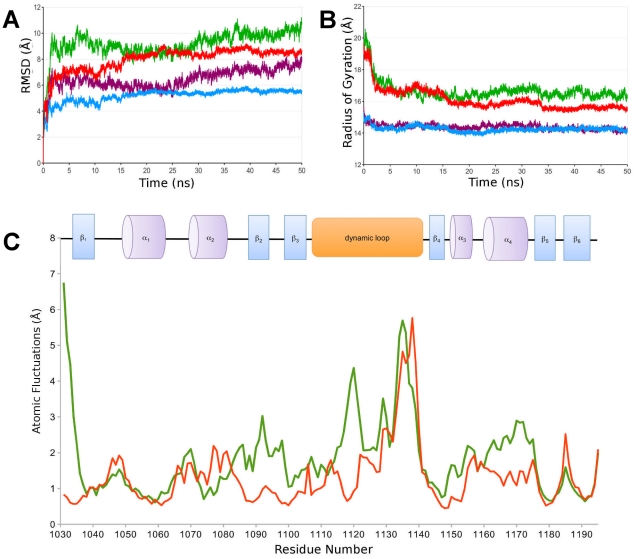
Analysis of the N-domain plasticity along MD simulations for WT and WT-Mg-ATP systems. (A) Root Mean Square Deviation (RMSD) of the C_α_atoms with the initial minimized structure used as reference for the 50 ns of MD simulations: whole Wild-Type protein of the N-domain (WT, green) and Mg^2+^-ATP complexed to the N-domain (WT-Mg-ATP, red). Purple and blue colors represent the RMSD of the C_α_ atoms calculated without taking into account the dynamic loop (A1114-T1143) for WT and WT-Mg-ATP system, respectively. All structures were previously fitted to the initial structure of the MD simulation considering the WT for all trajectories. (B) Radius of gyration of C_α_ atoms for WT and WT-Mg-ATP systems. Color scheme is the same as previously defined. (C) Atomic fluctuations of C_α_ atoms for WT (green) and WT-Mg-ATP systems (red).

Once the Mg^2+^ coordination site has been “validated”, a new ATP binding site could be proposed, taking into account all experimental and structural information already gathered (Table S1). The nucleotide was first positioned by molecular docking and was allowed to evolve along the MD simulation process to ensure proper Mg^2+^-ATP positioning through coordination of the phosphate tail. First, the N-domain was simulated through two different systems for 50 ns: WT and WT-Mg-ATP. Then, the structural validation of these two models was performed. The validation of the *in silico* models was done by comparing theoretical NMR chemical shifts to experimental data [22]. The Figure S4 shows an excellent agreement between calculated and experimental chemical shifts for C_α_ carbon atoms of the N-domains. A high correlation coefficient (R≥0.95) and a small dispersion of the scatter plot indicate that our 3D models are accurate. For the MD simulation, the stabilization of the Root Mean Square Deviation (RMSD) is achieved after 30 ns for the WT-Mg-ATP and WT systems as shown in Figure 3A. The global structure of the N-domain interacting with Mg^2+^-ATP shows a similar behavior to the nucleotide free N-domain. This is evidenced by the calculations of the RMSD: 9.9 Å and 8.6 Å for the RMSD of the N-domain alone (WT) and interacting with Mg^2+^-ATP (WT-Mg-ATP), respectively. The same result is obtained when the evolution of the RMSD is measured by excluding the “dynamic” loop (A1114-T1143). A similar trend is observed for the radius of gyration evolutions (Figure 3B). The R_g_ fluctuates around an average value of 16.5 Å for WT and 15.6 Å for the WT-Mg-ATP systems. Ramachandran plots show an increase in geometrical relaxation parameters for the structures of the N-domain between the initial and the representative structure of the last 20 ns of MD simulations (Figure S5). The initial WT N-domain structure has 73% of amino acids residues in the most favourable regions, whilst the representative structure of the last 20 ns of MD simulations reaches 84% for the WT and the WT-Mg-ATP systems.

Based on these MD preliminary results, we further investigated the Mg^2+^-ATP coordination site on the WT-Mg-ATP model. The position of the nucleotide for the representative structure of the last 20 ns of MD simulation is shown in Figure 2A. The magnesium ion is located near α_3_ and α_4_ helices (coordination helices). They bear the “DDE” motif and are located close to the glycine loop (^1099^GXGXG^1103^). The Figure 2B shows an overall “picture” of the new binding site where the adenine part of the nucleotide is located near the glycine loop and the β­sheets “core” of the N-domain. A close view in the nucleotide-binding environment is shown (Figure 2C). The magnesium ion is hexacoordinated via an octahedral geometry by the amino acids of the “DDE” motif (E1152, D1167, D1171) and oxygen atoms of the β­/γ­phosphate group of ATP. For the ribose part, N1150, W1153 and C1100 are the main protein residues in close interaction. The hydrogen bond between the backbone of C1100 (C = O) and one of the hydroxyl groups of the ATP ribose moiety has a 22% lifetime. The adenine moiety interacts essentially with P1098, G1099 of the glycine loop. The N1150 side-chain also interacts with one nitrogen atom of the adenine moiety. The E1152 residue interacts with the ion through bidentate coordination (Figure S6). In the WT-Mg-ATP MD simulation, the Mg^2+^ coordination is stable as shown by the average Mg^2+^-O distance (Figure S6). For the oxygen atoms of the side-chains of the “DDE” motif (E1152, D1167, D1171) and for the part of ATP interacting with the ion, the Mg^2+^-O distance is close to 2 Å ([1.84 Å– 1.96 Å]). This is compatible with previous structural data available on magnesium binding in proteins [23]. Neither the residues surrounding the coordinating sphere nor water molecules of the bulk solvent were able to induce any perturbation of the Mg^2+^-ATP coordination throughout the duration of MD simulation. The stabilization of the RMSD obtained after 30 ns for both simulations and the structural validation allow a further analysis of the nucleotide binding impact on each region of the N-domain. The general trend is a decrease in the atomic fluctuations for several regions of the protein when Mg^2+^-ATP is bound to the N-domain (Figure 3C). It appears that, at least, the movements of three regions are influenced by the presence of the nucleotide: the poly-glycine loop, the “dynamic” loop and the “coordination helices”. The glycine loop that is located between the two β-strands (β_2_, β_3_) and the dynamic loop, shows a noteworthy stabilization in the presence of the nucleotide. For example, the presence of ATP decreases the fluctuation of residues 1091–1099 with P1098 being the most important affected residue (2.3 Å versus 0.6 Å without ATP). The ATP presence under the loop enforces the two β-strands to accommodate their relative position. A hydrogen bond network rearrangement is occurring between the two β-strands with an increase in plasticity of the β_2_, which demonstrates a high potential of structural accommodation. The most marked effect of the Mg^2+^-ATP binding on the motions of the protein is observed for the long dynamic loop (A1114-T1143). Mg^2+^-ATP interactions reduce substantially the motion of several residues i.e. E1117 (−1.8 Å), R1118 (−2.6 Å), P1119 (−3.2 Å), L1120 (−3.4 Å), S1122 (−2.5 Å). The fluctuation of the “coordination helices” is also decreased. Indeed, the most significant difference between the MD simulations with or without Mg^2+^-ATP is seen for T1170 (−1.8 Å) and E1171 (−1.4 Å).

### ATP binding site without Mg^2+^

To assess the effect of Mg^2+^ on ATP binding to the N-domain, MD simulation was performed without the ion (WT-ATP system). The absence of the ion induces conformational changes in the N-domain with a major rearrangement concerning the α_3_ and α_4_ helices that can be explained by a decreasing structural order through MD simulation results of WT-ATP system. For the WT-ATP system, the stabilization of RMSD occurs after 20 ns (Figure S7A).

Computational results show that in absence of Mg^2+^, the N1150 is still in the vicinity of ATP molecule and interacts in this case (WT-ATP) with the phosphate moiety (O-Pγ) through hydrogen bond interaction (2.11 Å). Instead of interacting with Mg^2+^ ion (WT-ATP-Mg system), the D1171 amino acid forms a strong hydrogen bond with the hydroxyl group of the ribose moiety in absence of Mg^2+^ (WT-ATP system) (Figure S7B). In addition, the adenine moiety is encapsulated by M1035, P1098, I1102, I1138, G1149, M1174, I1180, and I1194 residues defining a hydrophobic pocket. Moreover, the amine group of adenine moiety forms hydrogen bond with carbonyl backbone of C1100 amino acid (2.87 Å). Molecular modeling results are confirmed by experimental data showing that ATP is able to bind the N-domain without the magnesium ion (Table 1). However, the affinity of the N-domain for ATP is 45% lower when Mg^2+^ is absent. All the above results suggest a more complex interaction between the N-domain and ATP than it has been previously suggested and reinforces the validity of a new ATP binding mode in absence of Mg^2+^ ion.

### Impact of the “dynamic loop” motion on Mg^2+^-ATP binding

As evidenced in the dynamic study of the Wild Type N-domain (WT), the motion of the long loop located between A1114 and T1143 is influenced by the presence of Mg^2+^-ATP. This striking change of motion suggests a possible contribution of this loop in ATP binding. Located between β_3_ and β_4_ (Figure 3B), this loop is only present in human P-ATPases such as ATP7B and ATP7A. The latter is a copper transporter highly homologous to the ATP7B protein, implicated in Menkes disease [24]. This explains why the role of this loop remains unknown besides an increasing numbers of structural studies actually available for these enzymes. According to the results obtained for the atomic fluctuations of the long loop, further investigations have been made to understand the role of this peculiar loop in the ATP7B function. First, the effect of the loop on the nucleotide binding was evaluated experimentally by the measurement of the K_d_ for a mutant protein with a shortened loop (deletion of residues 1121–1137). Unexpectedly, the absence of this loop suppresses the binding for both ATP and Mg^2+^-ATP (see Table 1). In order to understand these results and to elucidate furthermore the role of this loop in the N-domain dynamic response and its influence on the nucleotide binding process, one additional MD simulation has been performed. For this purpose, a new 3D model was built with the long “dynamic” loop substantially shortened (Shortened Loop SL, cf. Methods). No alteration of overall protein folding has been noticed, compared to WT-ATP-Mg model. The RMSD obtained for the SL-Mg-ATP system shows stabilization after 20 ns of MD simulations (Figure S8A). The Mg^2+^-ATP binding site is altered in absence of the whole loop with a re-organization of the magnesium coordination sphere (SL-Mg-ATP system). D1167 from the “DDE” motif is evicted and replaced by a water molecule (Figure S8B). The coordination helices loose their structural integrity. This allows the nucleotide to change its relative position concerning the coordination helices and the poly-glycine loop. It goes further “inside” the β-sheet core of the protein composed of a hydrophobic groove (I1119, I1180, I1194). The phosphate part of ATP is stabilized via an interaction with R1156. The adenine moiety looses its main interactions. These preliminary data on the loop role suggest interdependence between the motions of the dynamic loop and the Mg^2+^-ATP binding site via a possible affinity modulation of the Mg^2+^-ATP/N-domain complex.

## Discussion

Like in the case of WD, access to *in silico* technologies for studying the pathophysiology of rare genetic disorders, bring additional arguments for facilitating the diagnostic and the prognostic of diseases that are sometimes difficult to establish. A prerequisite for the study of the impact of point mutations on a protein structure is to understand its natural structural properties like folding, atomic motions, conformational changes, interaction with cofactors, etc. In the present work, the focus was the identification of the Mg^2+^-ATP complex binding site of the N-domain (WT). The scientific literature is abundant on P-ATPases. Many studies are focused on their catalytic cycle with an emphasis on the phosphorylation step [25] and the general role of ATP [20]. Less attention has been paid to the nucleotide-binding step. Structural studies have been published using molecular modeling tools to decipher at the molecular level the interactions between the protein and the nucleotide. For ATP7B, the first molecular modeling study was anterior to the publication of the NMR structure of the N-domain [15]. The putative three-dimensional (3D) model of the N-domain was obtained by homology modeling using the Sarcoplasmic Reticulum Ca^2+^-ATPase (SERCA) as a structural template. The study did not lead to significant results concerning the ATP binding process. The low primary sequence homology between the N-domain of ATP7B and SERCA (20%) led to a poorly accurate 3D model with two putative ATP binding configurations proposed in the same protein without any presence of Mg^2+^. Most of all, no experimental validation of the molecular interactions has been performed.

For the other members of the closely related P-ATPases, no molecular dynamics study of the nucleotide coordination is yet available. An NMR structure has been recently solved for the N-domain of ATP7A in the nucleotide bound (without Mg^2+^) and nucleotide free forms [26]. An observation of the ATP-bound structure (PDB Id. 2KMX) shows an overall domain organization similar to the ATP7B N-domain. However, the topology of the ATP7B nucleotide-binding site is rather different especially for the phosphate groups. Both proteins share key residues: P1098 (P1087 for ATP7A), G1099 (G1116), G1101 (G1118), N1150 (N1184). Another key element is the sequence conservation in both N-domains of the residues belonging to the “DDE” motif responsible of the Mg^2+^-ATP coordination in the WT-Mg-ATP model: E1157 (E1186), D1167 (D1201), and D1171 (D1205). The absence of Mg^2+^ in the ATP7A NMR structure could explain the differences observed in the nucleotide environment of two proteins. The possible modification of ATP conformation in presence of Mg^2+^ has been briefly evoked in the NMR study of the ATP7B N-domain [11] suggesting the existence of two different binding “sites” with different affinities for ATP. This hypothesis could explain the difference of the experimental K_d_ obtained for the WT in magnesium free or bound form (Table 1).

In order to challenge this assumption, MD simulations have been performed for the wild type ATP7B N-domain in interaction with either ATP or Mg^2+^-ATP (WT-ATP and WT-Mg-ATP systems). Indeed, ATP alone could accommodate its position by migrating towards a hydrophobic groove after the removal of the magnesium ion. The charge repulsion between phosphate tail of ATP and acidic side-chains of the residues belonging to the “DDE” motif of the Mg^2+^ coordination sphere could help ATP to shift. Two binding modes could exist in the N-domain whether the ion is present or not. The first binding mode needs Mg^2+^ for ATP binding to be tightly stuck to the N-domain, the second being a transitory binding mode before the migration of the nucleotide towards the P-domain. Indeed, the γ-phosphate hydrolysis is thus not possible for phosphorylation of the P-domain since it is engaged in the ion coordination through its oxygen atoms. For proper P-domain phosphorylation, ATP has to be separate from Mg^2+^. This transition might be possible through a conformational change of the N-domain (see below for Discussion about the dynamic loop) since the N-domain and Mg^2+^-ATP complex are stuck together through very strong electrostatic interactions. It would be interesting to test this hypothesis with a 3D model of N-/P-domains together to study the whole process from the nucleotide-binding step to the phosphyralation. This hypothesis is in agreement with the fact that Mg^2+^-ATP complex has to change its coordination when approaching the P-domain in order to allow its phosphorylation at D1027, as evidenced in a previous study concerning the SERCA structure solved in presence of ATP and Mg^2+^ [27]. A recent study of the CopA protein, a bacterial homolog of ATP7B, clearly brings a bundle of arguments in the favor of our hypothesis. In the crystal structure of the protein solved with an ATP analogue in presence of the magnesium ion [28], the nucleotide is bound in “sandwich” between the N- and P-domains nearby H1069 but not directly interacting with, and associated on the P-domain side with a hexacoordinated Mg^2+^ ion. It is interesting to note that there is no equivalent “DDE” triad in the N-domain of the CopA. More recently, a nearly complete X-ray structure has been published for this protein [29]. The presence of a magnesium ion near the DKTG motif at the interface of the P- and the A-domain support our hypothesis and could help to elucidate the full transfer of Mg-ATP from N- to P-domain during the acyl-phosphorylation process.

The previously published structural studies on the N-domain have neglected the role of the Mg^2+^ ion, although experimental evidences have pointed out the major role it plays in the nucleotide-binding mode of different P-ATPases. An original experimental protocol was used to study the ATP binding site of the Na^+^/K^+^ ATPase with Fe^2+^ ions capable of replacing Mg^2+^ [30]. A direct interaction was proposed between an ATP-Fe^2+^ complex and the aspartic acid side-chains of the “VADGA” motif (N-domain). Similar results for the SERCA have been confirmed by the interactions of residues of the N-domain and Mg^2+^-ATP [31]. The results obtained in the present study prove the essential role of the ion in the nucleotide binding to the N-domain as it accounts for a substantial part of the binding affinity. Theses results are in agreement with the previous experimental study published by Morgan *et al.* [17]. Indeed, a significant increase in affinity was measured with similar results for the K_d_: 75.30 µM (±3.62) for ATP alone to 57.00 µM (±3.00) for the Mg^2+^-ATP complex (Table 1). It was concluded surprisingly that the nucleotide binding to the N-domain is a magnesium-independent event despite contradictory experimental results. The reason was that no high-affinity binding has been observed in presence of N-domain and magnesium alone. Unfortunately, this publication has been often referred for justifying the lack of Mg^2+^ participation in ATP binding. Several other arguments sustain that Mg^2+^ always helps nucleotide to bind optimally to the protein target. It is well known that Mg^2+^ is essential for the catalytic activity of a wide range of enzymes using a nucleotide as cofactor (kinases, G-proteins, polymerases, etc, for review see reference [32]). In addition, the very low intracellular proportion of ATP in the free form, the cytosolic abundance of Mg^2+^ (∼10^−3^ M) and the important stability of the Mg^2+^-ATP complex (K_a_≈10^4^ M) explain why this complex is the major active form of the nucleotide [33]. This has been confirmed in muscle tissues where Mg^2+^-ATP accounts for 90% of total ATP [34]. The position of the ion in coordination with β­ and γ­phosphate oxygen atoms protects the nucleotide from hydrolysis. This fundamental role of the magnesium in binding sites is achieved through a hexacoordination under an octahedral geometry.

Our results show noticeable disparities with previously published work concerning the ATP binding site. A major difference concerns H1069 residue that has been shown to bind directly ATP without considering the role of the magnesium ion [11], [16]. H1069 residue has been proposed to participate directly in ATP binding since the H1069Q mutation occurs frequently in WD. Its participation in ATP binding is not obvious and has to be reconsidered for both modeling and binding experiments. Indeed, the distance between ATP and H1069 is about 9–10 Å for the WT-Mg-ATP system that is not compatible with a direct participation of the histidine in the binding site. Similarly, the simulation of WT-ATP model reveals a distance between ATP molecule and H1069 about 17 Å (distance in the representative structure) that indicates no direct participation in the ATP vicinity. Experiments confirmed this conclusion. The binding measurement repeated eight times for the H1069Q mutant is not different from the WT protein in both magnesium free and bound conditions (Table 1). Previous experimental results suggest a perturbation of ATP binding with a decrease in ATP affinity even though results are not reproducible. The first experimental measurement for the H1069Q mutant performed by isothermal titration calorimetry reported a substantial decrease in ATP binding affinity (K_d_ was estimated around 1200 µM) [17]. No alteration of the protein folding was reported. Then, Rodriguez-Granillo *et al.* obtained with the same method different results for the H1069Q mutation with only a two-fold decrease in affinity (K_d_ increase) as compared to the WT-ATP (344 µM for H1069Q and 166 µM for the WT) [16]. Moreover, the role of the magnesium ion was not considered in the binding experiments for the mutant. Such contradictory results leave the question amid controversy concerning the direct participation of the H1069 residue to the nucleotide binding. Another hypothesis has been proposed to explain the impact of the H1069Q mutation on the protein function. It was reported a total loss of phosphorylation of the conserved catalytic aspartic acid residue located in the P-domain [35]. This was observed without any alterations of the folding. This result is in agreement with the present work where the H1069 is far from the nucleotide-binding site and located close to the N-/P-domains interface. Similarly, Kenney and Cox reported that H1069Q mutation impacts on membrane expression due to the accumulation of the protein in the Golgi apparatus or in the endoplasmic reticulum [4]. All the above observations reinforce our findings.

Based on these fundamental data combined with *in silico* and structural biology techniques, we succeeded to clearly define the Mg^2+^-ATP N-domain binding site. Mg^2+^-ATP binding stabilizes the N-domain and reduces its internal atomic motions, particularly for the poly-glycine loop, the dynamic loop region and the N-terminal extremity of the protein. This latter observation remains to be clarified. This terminal part in our models evolves freely, whilst in the structural context of the whole ATP7B protein, it is buried and connects the N- and P-domains. The N-terminal extremity moves towards the nucleotide and close to the glycine-rich loop. That is not the case in the nucleotide free simulation of the WT system. The flexibility of this region had already been incriminated in different structural studies as responsible for the rotation of the N-domain towards the P-domain, a necessary conformational change in the phosphorylation step of the catalytic cycle of these enzymes [36], [37]. In addition, in the full protein, this N-terminal extremity bears the “DKTG” consensus motif of the P-ATPases suggesting that a possible local conformational change could regulate the catalytic activity of the enzyme. Let us bear in mind that all above conclusions are made on 3D models using the N-domain as a template. It would be interesting to study the impact of the nucleotide effect and the role of the long “dynamic loop” on the protein motions on the whole N-/P-domains.

**Figure 4:**
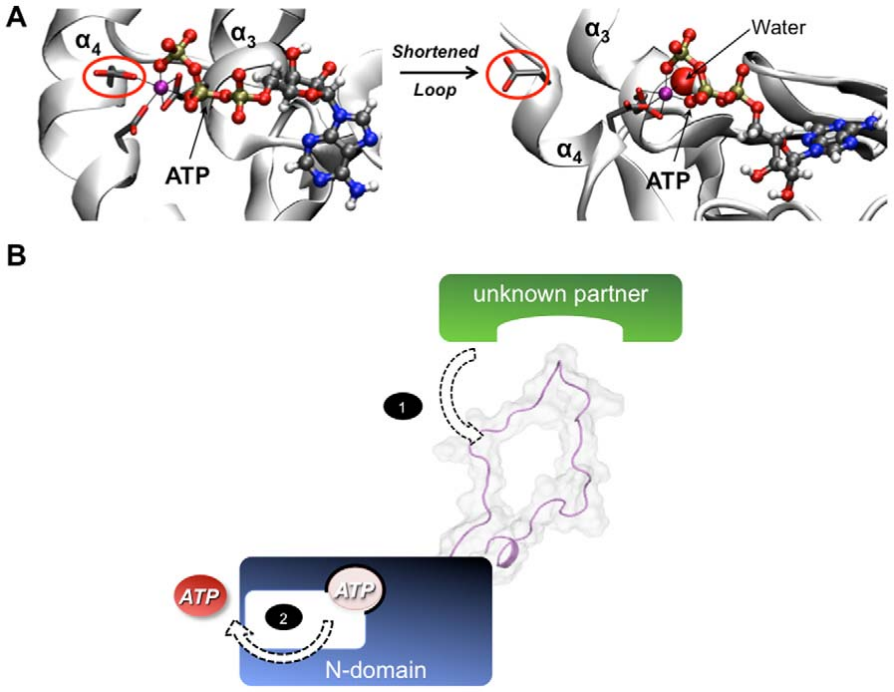
Implication of the flexible loop in ATP binding. (A) Closest trajectory snapshot of the representative structure for the whole and shortened N-domain (cartoon representation) for the WT-Mg-ATP (left) and SL-Mg-ATP (right) systems. The amino acid D1167 is circled in red and side-chains of the “DDE” motif are presented in sticks. ATP and water molecules are shown in ball and stick and VdW, respectively. (B) Schematic proposition of allosteric mechanism promoted by the dynamic loop for the nucleotide release process. The first step of the dynamic response of the loop implies a constraint applied by an unknown partner. The second step is the perturbation of the binding of ATP with the N-domain.

We have also shown that shortening the dynamic loop results in a perturbation of ATP binding such as an alteration of the interactions in the Mg^2+^ environment. Indeed, the octahedral Mg^2+^ coordination is modified with the removal of D1167 residue, which is replaced by a water molecule (Figure 4A). This modification could be due to the perturbation of the α_3_/α_4_ helices. The presence of the loop only in ATP7B among all the ATPase protein family suggests an original participation of this loop to the allosteric regulation of ATP binding. Indeed, it could be argued that the reduction of this loop motions could facilitate phosphorylation of the ATP7B P-domain by inducing a decrease in the Mg^2+^-ATP binding force to the N-domain. Moreover, a possible unknown protein partner could interact with the loop protruding in the cytosol, out of the Golgi membrane (Figure 4B). All the above data open up a new way for additional experimental investigations for characterizing this putative dynamic loop partner. It could be an unknown protein or another domain of the ATP7B protein. Indeed, a direct interaction between the complex formed by association of the N- and the P-domains and the N-terminal Copper-Binding Domain has been demonstrated [38].

In summary, we provided a clear definition of the topology and localization of the Mg^2+^-ATP binding site by using a careful *in silico* protocol. Through the N-domain protein, the “DDE” motif (E1152, D1167, D1171) is able to coordinate the ion via an octahedral geometry (Figure 2). The absence of the Mg^2+^ ion led to a topology description of a transitory binding mode that could play an important role before the phosphorylation of the P-domain. Computational results such as the intervention of the ion or the role of the dynamic loop have been confirmed experimentally. The binding measurements have shown that the nucleotide is able to bind the N-domain with a submicromolar affinity in presence of Mg^2+^ ion. Most of all, the possible allosteric role by the long dynamic loop in the N-domain Mg^2+^-ATP binding/release was put forward. All above results pave the road for additional investigations of the impact of mutations on the folding and function of the N-domain.

## Methods

### Structural analysis of Mg^2+^-nucleotide binding site

In order to find the ATP7B N-domain Mg^2+^-ATP binding site, a database search was done on the MSD motif database of the European Bioinformatics Institute [39]. It contains 19 042 different bonds interacting between amino acids and Mg^2+^ ion. The results are ranked for every amino acid participating to Mg^2+^ coordination in a decreasing order of occurrence probability (Figure S1). Then, based on this classification, a pharmacophore-based approach [39] is used to find a putative magnesium-binding site in the N-domain of ATP7B. The 3D structure of the N-domain is inserted on 3D grid of points with 1 Å between each point. Its surface is then mapped using a pharmacophore of an ideal Mg^2+^ coordination geometry (Figure S2). Each pharmacophoric point was designed according to functional chemical groups of the amino acid side-chains involved in the Mg^2+^ ion coordination. As an example of the most represented coordinating residues, it contains different pharmacophoric points (Asp, Glu, etc.). The definition of pharmacophoric points includes a sphere centered on Mg^2+^ with C_α_ atoms bearing active points. The sphere radius is adjusted depending on the volume of every atom of the side-chains (Asp, Glu, etc.), the number of residues to be included and their relative orientation. The 6 Å diameter of the sphere takes into account the volume of the spheres for the oxygen atoms and Mg^2+^ based on their respective Van der Waals radii (1.52 Å and 1.71 Å). This is compatible with the fact that experimental Mg^2+^-O distance is rarely superior to 3 Å. The mapping procedure was repeated several times by varying systematically the nature of each pharmacophoric point and by respecting the occurrence probability of each amino acid obtained by the previous ranking procedure with an emphasis on acidic residues.

The validation of the Mg^2+^ nucleotide coordination site was achieved by performing a careful 3D visual analysis of 3D structures containing magnesium, ATP or analogues available in the Protein Data Bank (PDB). First, 72 proteins containing a nucleoside triphosphate hydrolase P-loop structural motif were studied (SCOP classification [40]). Next, the analysis was extended to 97 proteins bearing a Rossman fold (CATH classification [41]). Finally, we expanded our PDB “scan” to 314 matching structures sharing the same nucleotide binding function as the N-domain (Gene Ontology term 166). Every nucleotide-binding pattern was carefully analyzed with the help of PyMOL visualization program [42].

### Molecular dynamics simulations

The NMR “model 1” structure of the N-domain of the ATP7B protein (PDB code 2ARF) was used as a starting structure for molecular dynamics (MD) simulations with the help of the GROMACS molecular dynamics package [43]. The WT and shortened N-domain (Shortened Loop: SL) isolated species as well as complexed with either ATP (WT-ATP) or Mg^2+^-ATP (WT-Mg-ATP) were simulated. The initial position of the nucleotide was obtained from molecular docking using the WT model obtained from MD with the magnesium ion positioned near the “DDE” motif. Conformational space for ATP positioning was performed taking into account the residues highlighted from experimental and structural informations (Table S1). Then, calculations were done using AUTODOCK 4.2 [44]. 200 docking runs with Lamarckian Genetic Algorithm as a conformational search engine were done and results were clustered with a RMSD tolerance of 2 Å. The lowest energy conformation of the most populated cluster was then considered for following MD simulations.

All studied systems were prepared using Ambertools package [45]. The protein and the magnesium ion (Mg^2+^) were described with the FF03 force field for proteins [46], [47]. ATP parameters were obtained from GAFF force field [48] and additional revised parameters [49], [50]. Topology and coordinates files obtained from tLEaP were then converted to GROMACS file format using Acpype [51] python tool based. Each model was solvated in a rectangular water box with a 12 Å layer of TIP3P water molecules. The protein residues were considered at neutral pH. Thus, HIS residues were kept in the default neutral protonation state (HID, with hydrogen on the delta nitrogen) in Amber FF03. Electro neutrality was achieved through the addition of the appropriate number of Na^+^ counter ions. Energy minimization protocol included 5000 cycles of Steepest Descent algorithm with convergence criteria of 500 kJ.mol^−1^.nm^−1^. The electrostatic non-bonded interactions were computed with a 12 Å cut-off and particle-mesh Ewald method [52] to treat long range interactions. Lennard-Jones potential was described with a continuously smoothing cut-off from 10 to 12 Å. A constant temperature/pressure simulation with a 1 fs time step under leap-frog algorithm for integrating Newton's equations of motion was performed. A total of 50 ns simulations were done for each system at 310 K in the NPT ensemble (constant number of particles, pressure, and temperature) with temperature velocity rescaling coupling [53] and at 1 atm pressure with Berendsen isotropic coupling [54]. The study of the impact of the large dynamic loop (residues A1114 to T1143) on the N-domain/Mg^2+^-ATP complex was investigated by shortening the loop (lacking residues 1121–1137) and by linking residues 1120 and 1138 to give SL-Mg-ATP system. The geometrical quality of the different representative 3D structures throughout the simulations was assessed by Ramachandran plots generated by the PROCHECK program [55] (Figure S5). For protein-ligand interactions studies and other analyses, representative structures were extracted using the closest frame from the representative structure over the last 20 ns of each trajectory. Hydrogen bonds have been considered with precise geometrical parameters: 3.2 Å donor-acceptor distance cutoff and a 30° angle cutoff.

### Protein expression and purification

To analyze the pertinence of the Mg^2+^-ATP binding site, functional profiles of engineered N-domains of ATP7B (amino acid residues V1036-D1196 of the full-length protein) were determined. The corresponding cDNA region was PCR-amplified and subcloned as previously described [17]. Mutations were introduced using the QuickChange site-directed mutagenesis kit (Stratagene®) and verified by DNA sequencing (3130 Genetic Analyzer, Applied Biosystems). Wild type or mutated cDNAs were then expressed and the corresponding proteins were purified. To avoid transcriptional bias, cell lines expressing comparable amounts of RNA from cDNA were selected by Taqman analysis.

### Nucleotide binding experiments

Surface Plasmonic Resonance was used to determine the binding properties of WT and several mutants of N-domain with ATP and Mg^2+^-ATP. The ATP7B/ATP and ATP7B/ATP, Mg^2+^ interactions were determined by surface plasmon resonance (SPR, BIAcore AB, Uppsala, Sweden) at 25°C using 10 mM sodium phosphate (pH 7.40) containing 150 mM NaCl as running buffer (phosphate-buffered saline, PBS). The flow rate was 5 µL/min during immobilization. Purified Wild Type and mutated ATP7B were covalently immobilized onto the hydrophilic carboxymethylated dextran matrix of the sensor chip CM4 previously activated with 35 µL activation solution, as described by the manufacturer. We used 10 µL (1 mM) of ATP or ATP +12 mM MgCl_2_ (Sigma) dissolved in 10 mM sodium acetate (pH 5.00). A reference surface was subjected to the same procedure, but with no protein. The chip was blocked using 35 µL of blocking solution (1 M ethanolamine hydrochloride, pH 8.50). We then injected the ATP or ATP+Mg^2+^ solution and collected data at a flow rate of 30 µL/min. Data were analyzed by nonlinear curve fitting using the manufacturer's software. Each binding experiment was repeated several times (n = 6–8) under both conditions.

### Spectral characterization of theoretical models

The experimental validation of the generated 3D models was performed by comparison between the available NMR chemical shifts of the N-domain and “theoretical” chemical shifts calculated from MD trajectories. The various assigned experimental chemical shifts were extracted from the BioMagResBanK (entry number 6914). Apart from general NMR parameters such as relaxation times (T_1_, T_2_), the chemical shifts are not available directly from simulation data. The SPARTA program [22] predicts protein backbone chemical shifts (^15^N, ^1^H^N^, ^1^H_α_, ^13^C_α_, ^13^C_β_, ^13^C′) from a PDB file. According to the elevated Pearson's correlation coefficient (R = 0.91) between experimental and SPARTA predicted chemicals shifts calculated for ^13^C_α_ atoms, we decided to use these atoms to evaluate the correlation between experimental and calculated NMR shifts from our 3D models.

## Supporting Information

Figure S1 Bar chart showing the proportion of the different amino acids involved in Mg^2+^ coordination (source EBI). The percentage of magnesium ion interacting with either an aspartic or a glutamic acid is shown.
(DOC)

Figure S2 N-domain screening procedure used for identification of the Mg^2+^ binding coordination site. The N-domain is represented in cartoon, aspartic and glutamic acids side-chains in sticks. The pharmacophore presented here is an example of a fit obtained with Mg^2+^ and three amino acids (Asp, Asp, Glu). The result of the protein surface mapping is green circled and two important regions of the protein known to play a role in ATP binding are shown with black arrows (G1149-N1150, GxG motif).
(DOC)

Figure S3 Examples of the Mg^2+^ coordination environment in different ATP-binding proteins: (A) Human nicotinamide riboside kinase, (B) yeast mitochondrial F1-ATPase, (C) GlcV, bacterial ABC-ATPase of the glucose ABC transporter, (D) ATPase domain of the bovine heat-shock cognate protein. Each 3D structure is identified with its PDB Id code. The ATP (or analogue) is colored in gray, the magnesium sphere in purple.
(DOC)

Figure S4 Correlation plots between experimental and calculated NMR chemical shifts obtained from different structures for the ^13^C_α_ carbon atoms of the N-domain: (A) Initial structure available in the PDB, (B) Structure of the representative frame of the last 20 ns of 50 ns MD trajectories of the WT-Mg-ATP system and (C) of the WT system. Predictions of the chemical shifts were obtained using the SPARTA software (see Methods).
(DOC)

Figure S5 Ramachandran plots of the N-domain monitoring the structural components of the models used throughout the molecular dynamics (MD) simulations. Initial structure after the minimization step (upper plot), structure of the representative frame of the last 20 ns of 50 ns MD trajectories for WT (left) and WT-Mg-ATP systems (right). The percentage of residues found in the most favorable regions of the diagram is indicated in a purple rectangle for each model.
(DOC)

Figure S6 Geometrical details of the octahedral Mg^2+^ coordination in the nucleotide-binding site. The trajectory snapshot of the system WT-ATP-Mg represents atoms in close vicinity of the ion (magenta sphere). The representative structure of the last 20 ns of MD simulations is shown with the octahedral Mg^2+^ coordination in the nucleotide-binding site. The distances (Å) related to the octahedral coordination of the Mg^2+^ are the average values over the last 20 ns of the total dynamic simulation (50 ns).
(DOC)

Figure S7 MD analysis and alternative binding mode of the N-domain in absence of Mg^2+^ for the WT-ATP system. (A) Plot of the Root Mean Square Deviation (RMSD, in Å) of the C_α_,atoms along the 50 ns of MD simulation for the WT-ATP system. (B) Closer trajectory snapshot of the representative structure along the last 20 ns of the 50 ns duration. Protein (WT), ATP molecule, and side-chain residues are represented in cartoon, ball and stick, and tube, respectively. Hydrogen bonds are shown in doted line.
(DOC)

Figure S8 MD analysis and binding mode of the N-domain where the dynamic loop (A1114-T1143) has been shortened for the SL-Mg-ATP system. (A) Plot of the Root Mean Square Deviation (RMSD, in Å) of the C_α_atoms along the 50 ns of MD simulation for the SL-Mg-ATP system (red). (B) Closer trajectory snapshot of the representative structure along the last 20 ns of the 50 ns duration. Protein (shortened dynamic loop, SL), ATP and water molecules, magnesium atom and side-chain residues are represented in cartoon, ball and stick, VdW, and tube, respectively. Hydrogen bonds are shown in doted line.
(DOC)

Table S1 Structural and experimental information used to propose an initial binding site for ATP in the molecular dynamics simulations. The residues highlighted in yellow green belong to the glycine loop and light blue for the coordination helices.
(DOC)
