# Supplementary material for: Elucidation of the ATP7B N-Domain Mg2+-ATP Coordination Site and Its Allosteric Regulation
Source: PLoS One. 2011 Oct 27;6(10):e26245. doi: 10.1371/journal.pone.0026245 (PMC3203118; doi:10.1371/journal.pone.0026245)
Supplement: Figure S1 — Bar chart showing the proportion of the different amino acids involved in Mg2+ coordination (source EBI). The percentage of magnesium ion interacting with either an aspartic or a glutamic acid is shown. (DOC) [file pone.0026245.s001.doc]

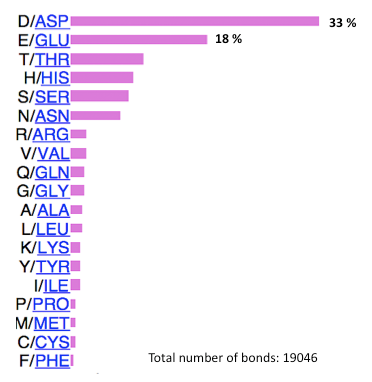


**Figure S1.** Bar chart showing the proportion of the different amino acids involved in Mg^2+^ coordination (source EBI). The percentage of magnesium ion interacting with either an aspartic or a glutamic acid is shown.
