# Supplementary material for: Elucidation of the ATP7B N-Domain Mg2+-ATP Coordination Site and Its Allosteric Regulation
Source: PLoS One. 2011 Oct 27;6(10):e26245. doi: 10.1371/journal.pone.0026245 (PMC3203118; doi:10.1371/journal.pone.0026245)
Supplement: Figure S2 — N-domain screening procedure used for identification of the Mg2+ binding coordination site. The N-domain is represented in cartoon, aspartic and glutamic acids side-chains in sticks. The pharmacophore presented here is an example of a fit obtained with Mg2+ and three amino acids (Asp, Asp, Glu). The result of the protein surface mapping is green circled and two important regions of the protein known to play a role in ATP binding are shown with black arrows (G1149-N1150, GxG motif). (DOC) [file pone.0026245.s002.doc]

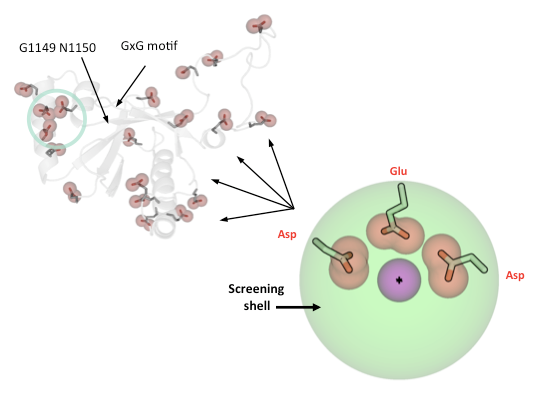


**Figure S2.** N-domain screening procedure used for identification of the Mg^2+^ binding coordination site. The N-domain is represented in cartoon, aspartic and glutamic acids side-chains in sticks. The pharmacophore presented here is an example of a fit obtained with Mg^2+^ and three amino acids (Asp, Asp, Glu). The result of the protein surface mapping is green circled and two important regions of the protein known to play a role in ATP binding are shown with black arrows (G1149-N1150, GxG motif).
