# Supplementary material for: Elucidation of the ATP7B N-Domain Mg2+-ATP Coordination Site and Its Allosteric Regulation
Source: PLoS One. 2011 Oct 27;6(10):e26245. doi: 10.1371/journal.pone.0026245 (PMC3203118; doi:10.1371/journal.pone.0026245)
Supplement: Figure S3 — Examples of the Mg2+ coordination environment in different ATP-binding proteins: (A) Human nicotinamide riboside kinase, (B) yeast mitochondrial F1-ATPase, (C) GlcV, bacterial ABC-ATPase of the glucose ABC transporter, (D) ATPase domain of the bovine heat-shock cognate protein. Each 3D structure is identified with its PDB Id code. The ATP (or analogue) is colored in gray, the magnesium sphere in purple. (DOC) [file pone.0026245.s003.doc]

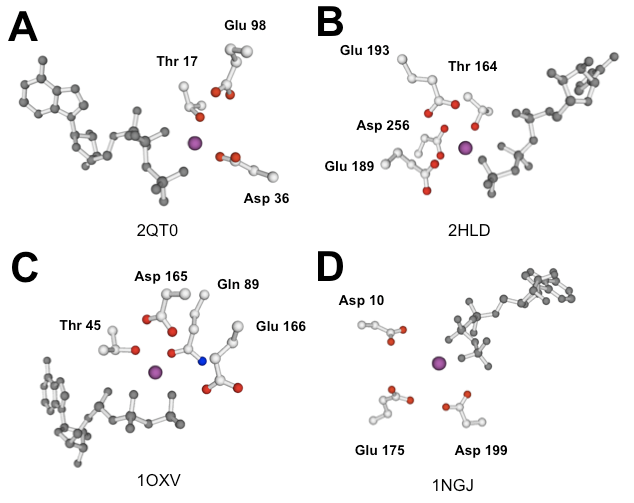


**Figure S3.** Examples of the Mg^2+^ coordination environment in different ATP-binding proteins: (A) Human nicotinamide riboside kinase, (B) yeast mitochondrial F1-ATPase, (C) GlcV, bacterial ABC-ATPase of the glucose ABC transporter, (D) ATPase domain of the bovine heat-shock cognate protein. Each 3D structure is identified with its PDB Id code. The ATP (or analogue) is colored in gray, the magnesium sphere in purple.
