# Supplementary material for: Elucidation of the ATP7B N-Domain Mg2+-ATP Coordination Site and Its Allosteric Regulation
Source: PLoS One. 2011 Oct 27;6(10):e26245. doi: 10.1371/journal.pone.0026245 (PMC3203118; doi:10.1371/journal.pone.0026245)
Supplement: Figure S4 — Correlation plots between experimental and calculated NMR chemical shifts obtained from different structures for the 13Cα carbon atoms of the N-domain: (A) Initial structure available in the PDB, (B) Structure of the representative frame of the last 20 ns of 50 ns MD trajectories of the WT-Mg-ATP system and (C) of the WT system. Predictions of the chemical shifts were obtained using the SPARTA software (see Methods). (DOC) [file pone.0026245.s004.doc]

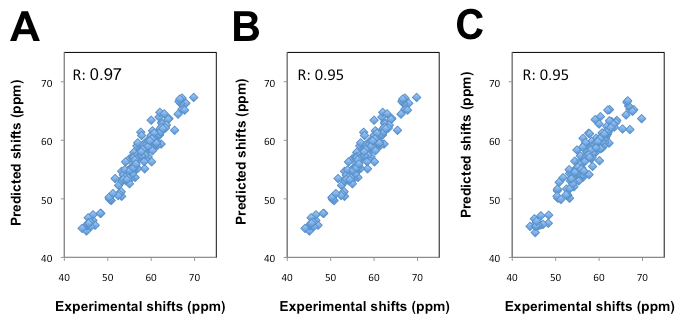


**Figure S4.** Correlation plots between experimental and calculated NMR chemical shifts obtained from different structures for the ^13^C_α_ carbon atoms of the N-domain: (A) Initial structure available in the PDB, (B) Structure of the representative frame of the last 20 ns of 50ns MD trajectories of the WT-Mg-ATP system and (C) of the WT system. Predictions of the chemical shifts were obtained using the SPARTA software (see Methods).
