# Supplementary material for: Elucidation of the ATP7B N-Domain Mg2+-ATP Coordination Site and Its Allosteric Regulation
Source: PLoS One. 2011 Oct 27;6(10):e26245. doi: 10.1371/journal.pone.0026245 (PMC3203118; doi:10.1371/journal.pone.0026245)
Supplement: Figure S5 — Ramachandran plots of the N-domain monitoring the structural components of the models used throughout the molecular dynamics (MD) simulations. Initial structure after the minimization step (upper plot), structure of the representative frame of the last 20 ns of 50 ns MD trajectories for WT (left) and WT-Mg-ATP systems (right). The percentage of residues found in the most favorable regions of the diagram is indicated in a purple rectangle for each model. (DOC) [file pone.0026245.s005.doc]

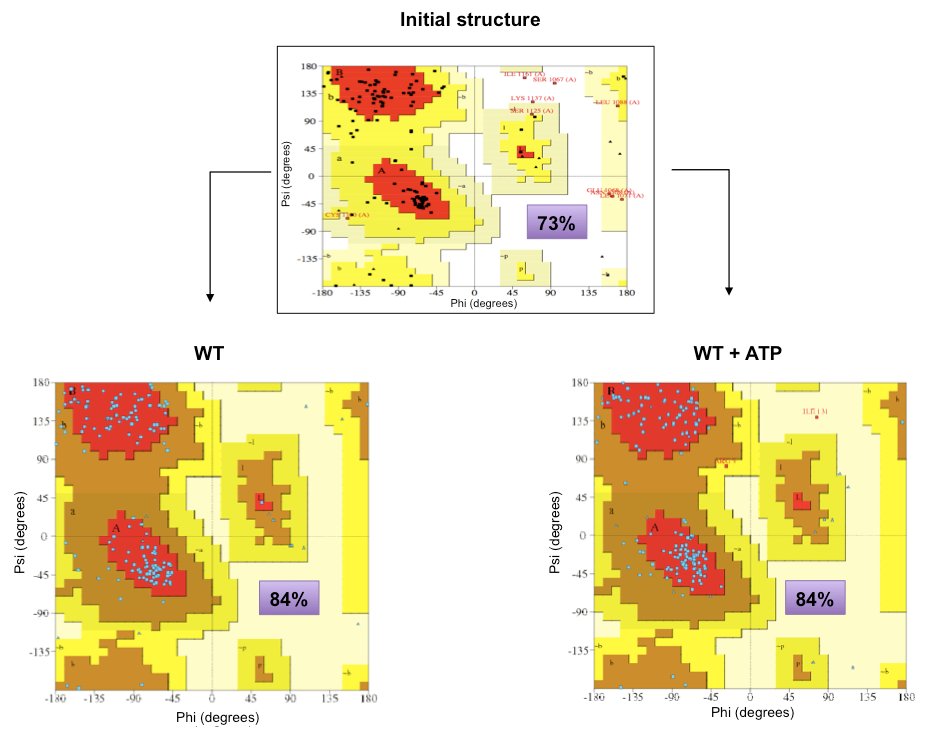


**Figure S5.** Ramachandran plots of the N-domain monitoring the structural components of the models used throughout the molecular dynamics (MD) simulations. Initial structure after the minimization step (upper plot), structure of the representative frame of the last 20 ns of 50 ns MD trajectories for WT (left) and WT-Mg-ATP systems (right). The percentage of residues found in the most favorable regions of the diagram is indicated in a purple rectangle for each model.
