# Supplementary material for: Elucidation of the ATP7B N-Domain Mg2+-ATP Coordination Site and Its Allosteric Regulation
Source: PLoS One. 2011 Oct 27;6(10):e26245. doi: 10.1371/journal.pone.0026245 (PMC3203118; doi:10.1371/journal.pone.0026245)
Supplement: Figure S6 — Geometrical details of the octahedral Mg2+ coordination in the nucleotide-binding site. The trajectory snapshot of the system WT-ATP-Mg represents atoms in close vicinity of the ion (magenta sphere). The representative structure of the last 20 ns of MD simulations is shown with the octahedral Mg2+ coordination in the nucleotide-binding site. The distances (Å) related to the octahedral coordination of the Mg2+ are the average values over the last 20 ns of the total dynamic simulation (50 ns). (DOC) [file pone.0026245.s006.doc]

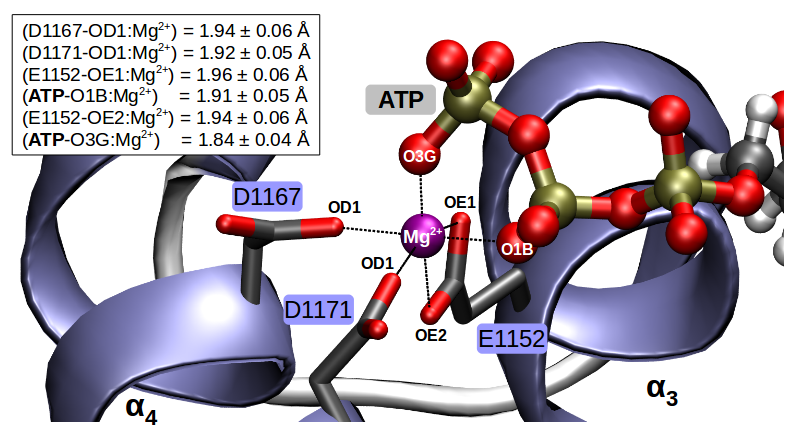


**Figure S6.** Geometrical details of the octahedral Mg^2+^ coordination in the nucleotide-binding site. The trajectory snapshot of the system WT-ATP-Mg represents atoms in close vicinity of the ion (magenta sphere). The representative structure of the last 20 ns of MD simulations is shown with the octahedral Mg^2+^ coordination in the nucleotide-binding site. The distances (Å) related to the octahedral coordination of the Mg^2+^ are the average values over the last 20 ns of the total dynamic simulation (50 ns).
