# Supplementary material for: Elucidation of the ATP7B N-Domain Mg2+-ATP Coordination Site and Its Allosteric Regulation
Source: PLoS One. 2011 Oct 27;6(10):e26245. doi: 10.1371/journal.pone.0026245 (PMC3203118; doi:10.1371/journal.pone.0026245)
Supplement: Figure S7 — MD analysis and alternative binding mode of the N-domain in absence of Mg2+ for the WT-ATP system. (A) Plot of the Root Mean Square Deviation (RMSD, in Å) of the Cα,atoms along the 50 ns of MD simulation for the WT-ATP system. (B) Closer trajectory snapshot of the representative structure along the last 20 ns of the 50 ns duration. Protein (WT), ATP molecule, and side-chain residues are represented in cartoon, ball and stick, and tube, respectively. Hydrogen bonds are shown in doted line. (DOC) [file pone.0026245.s007.doc]

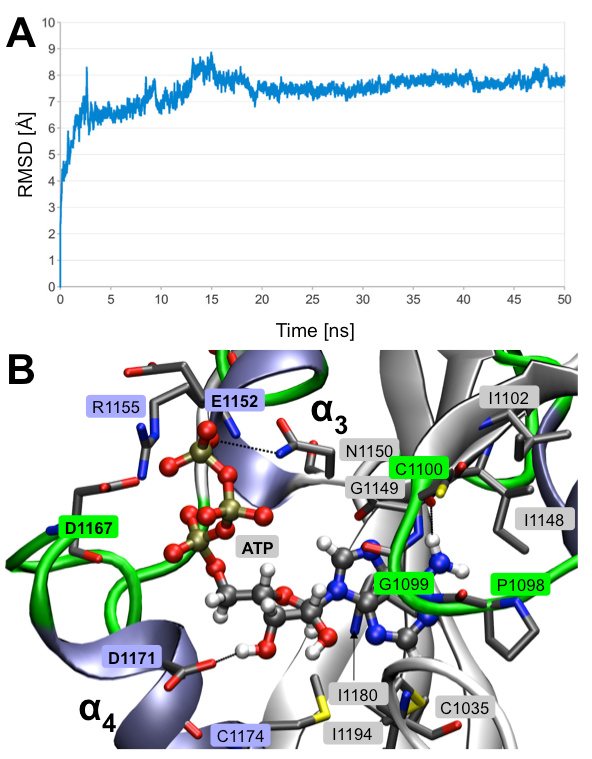


**Figure S7.** MD analysis and alternative binding mode of the N-domain in absence of Mg^2+^ for the WT-ATP system. (A) Plot of the Root Mean Square Deviation (RMSD, in Å) of the C_α_ atoms along the 50 ns of MD simulation for the WT-ATP system. (B) Closer trajectory snapshot of the representative structure along the last 20 ns of the 50 ns duration. Protein (WT), ATP molecule, and side-chain residues are represented in cartoon, ball and stick, and tube, respectively. Hydrogen bonds are shown in doted line.
