# Supplementary material for: Elucidation of the ATP7B N-Domain Mg2+-ATP Coordination Site and Its Allosteric Regulation
Source: PLoS One. 2011 Oct 27;6(10):e26245. doi: 10.1371/journal.pone.0026245 (PMC3203118; doi:10.1371/journal.pone.0026245)
Supplement: Figure S8 — MD analysis and binding mode of the N-domain where the dynamic loop (A1114-T1143) has been shortened for the SL-Mg-ATP system. (A) Plot of the Root Mean Square Deviation (RMSD, in Å) of the Cαatoms along the 50 ns of MD simulation for the SL-Mg-ATP system (red). (B) Closer trajectory snapshot of the representative structure along the last 20 ns of the 50 ns duration. Protein (shortened dynamic loop, SL), ATP and water molecules, magnesium atom and side-chain residues are represented in cartoon, ball and stick, VdW, and tube, respectively. Hydrogen bonds are shown in doted line. (DOC) [file pone.0026245.s008.doc]

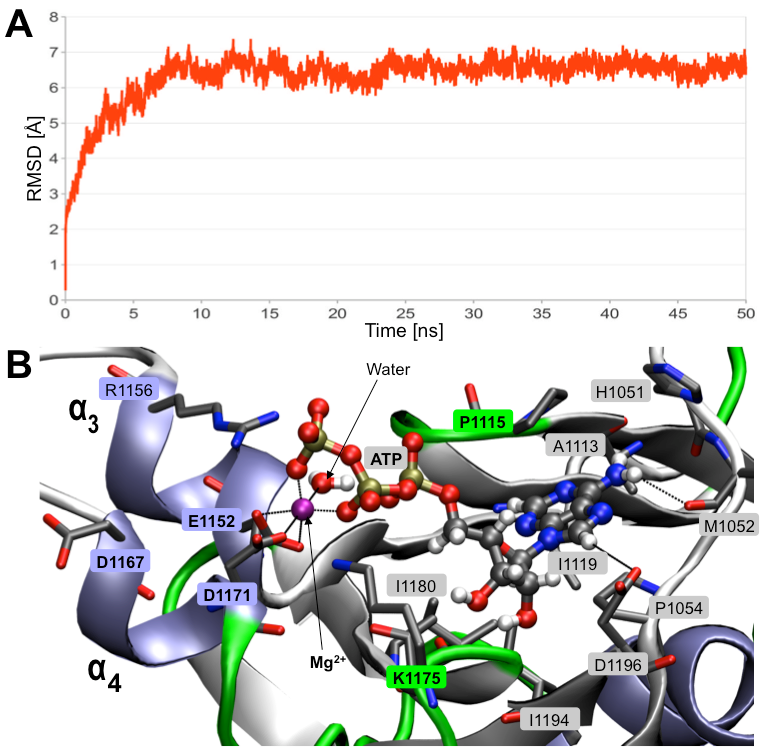


**Figure S8.** MD analysis and binding mode of the N-domain where the dynamic loop (A1114-T1143) has been shortened for the SL-Mg-ATP system. (A) Plot of the Root Mean Square Deviation (RMSD, in Å) of the C_α_ atoms along the 50 ns of MD simulation for the SL-Mg-ATP system (red). (B) Closer trajectory snapshot of the representative structure along the last 20 ns of the 50 ns duration. Protein (shortened dynamic loop, SL), ATP and water molecules, magnesium atom and side-chain residues are represented in cartoon, ball and stick, VdW, and tube, respectively. Hydrogen bonds are shown in doted line.
