# Supplementary material for: Elucidation of the ATP7B N-Domain Mg2+-ATP Coordination Site and Its Allosteric Regulation
Source: PLoS One. 2011 Oct 27;6(10):e26245. doi: 10.1371/journal.pone.0026245 (PMC3203118; doi:10.1371/journal.pone.0026245)
Supplement: Table S1 — Structural and experimental information used to propose an initial binding site for ATP in the molecular dynamics simulations. The residues highlighted in yellow green belong to the glycine loop and light blue for the coordination helices. (DOC) [file pone.0026245.s009.doc]

| **Residue** | **N-domain localization** | **ATP interaction** | **Origin** | **Reference** |
| --- | --- | --- | --- | --- |
| G1099 | glycine loop | ribose | Δδ_ATP_ (NMR chemical shift) | 11 |
| G1101 | glycine loop | adenine | Δδ_ATP_ (NMR chemical shift) | 11 |
| G1149 | - | adenine | NOE restraint | 11 |
| N1150 | - | ribose | NOE restraint | 11 |
| E1152 | coordination helices | phosphate | pharmacophore screening | this study |
| D1167 | coordination helices | phosphate | pharmacophore screening | this study |
| D1171 | coordination helices | phosphate | pharmacophore screening | this study |

**Table S1.** Structural and experimental information used to propose an initial binding site for ATP in the molecular dynamics simulations. The residues highlighted in yellow green belong to the glycine loop and light blue for the coordination helices.
